# Round the Hospitals

**Published:** 1916-03-18

**Authors:** 


### THE MATRONS' AND SISTERS' DEPARTMENT.

### Round the Hospitals.

We regret to report the death, on the 6th instant, of Principal Sister Harrower, of the County of London War Hospital, Epsom. She had three years' training at the Royal Infirmary, Edinburgh, and two years' fever training at Stratford-on-Avon. She was a private nurse for some time, and had been superintendent sister at Queen Mary's Hospital, Carshalton. She was highly respected, and had a uniformly kindly, cheerful, and tactful disposition. The funeral service took place on the 9th instant in the hospital church, and Sister Harrower was subsequently buried in the Epsom Cemetery, her funeral being attended by the officer commanding, Miss Thorburn (the matron), and most of the principal officers, military and otherwise. Sister Harrower will be greatly missed, for she was the first sister to be engaged at Epsom Hospital, and rendered invaluable help to the matron there in the organisation of the nursing staff, whose affections she readily won.

---

Reverting to the County Hospital Matron's request in our issue of March 4 on the subject of the laundry department, we are disappointed in not having received so far a communication from the sister-in-charge of the laundry at one of the large Scottish hospitals at Edinburgh, Glasgow, or Perth, to name only three where we have seen most excellent laundry work in operation. Will not the sister-in-charge come to the aid of the County Hospital Matron by setting down her experience of an efficient laundry and how to manage it? We have received one contribution from an English matron of great practical experience. Her view is opposed to that expressed by some authorities on the subject, for she is of opinion that if the machinery is suitable and in good order the cost of working the hospital laundry is considerably less than that of sending the linen out to a public laundry. Another advantage which she insists upon is that less linen is required because the washing is returned more quickly. There is less risk of things being lost, and it is a great convenience for the matron to have the service of clothing and linen to the wards under her direct control. This opinion raises many interesting points, upon which we invite a discussion, because they include matters which have an important bearing on efficiency from the matron's point of view. We hope at an early date to publish an article by a matron on the equipment and staff required for a laundry attached to a hospital with 120 beds.

---

The Military and Territorial Hospitals throughout have done original and good things in organising this new type of institution within the British Islands. We have before set forth the excellence of some hospital magazines and gazettes which have emanated from this source, and have had the privilege of reproducing some excellent illustrations from their pages. This week Miss E. O. Thomson, the matron of the 4th Scottish General Hospital, Stobhill, Glasgow, sends us an account of the first series of whist-drives for the wounded. These drives have been well organised and have proved a notable success. They were started by the generosity of the Glasgow Musical Artistes' Association, which presented a handsome silver cup for competition among the soldiers. Each ward selected its two best players and the competitors met in one of the wards in accordance with a time-table, and competed for the possession of the cup. To add to its attractions this cup is allowed to remain for one month in the ward which the winners represented. From start to finish the competition created genuine fun. The initial stage of selecting the ward champions made the inmates merry, and on the conclusion of the competition another evening was pleasantly spent in the ward of the victors, decorated for the occasion, where tea was served and the cup formally presented and placed in position. On the evening when the competition took place, Colonel Napier, with some other officers, attended. The competition was followed with keen interest, and the representatives of Ward 7A, who won the cup, were received with vociferous congratulations. Colonel Napier's tea was a happy thought on the part of the Commandant, and the music which accompanied it, and the good songs sung, were greatly enjoyed. We have much pleasure in publishing this account of a new departure, so far as our knowledge goes. It should contribute to the recovery of the wounded, by fitting them as speedily as possible for the arduous and patriotic duty which lies before them on their return to sound condition and perfect health.

---

Hospital thrift advocated by a ward sister is a subject of great importance, but all the responsibility does not rest with her. In many hospitals little is done to encourage economy in the wards. Sometimes sisters have the need for checking waste dinned into their ears till they begin to lose heart. The conscientious worker feels that, try as she may, she never reaches the goal, and some hospital authorities seem afraid to acknowledge when she succeeds for fear that she may relax her efforts. An old sister writes of this aspect as follows: "The nurses and probationers get tired of being reminded to put out the light in the linen cupboard, and the wardmaid is rude when she is found fault with for throwing away good pieces of bread. All this when a sister is tired may create the temptation to give up trying quite so hard and to let things go. Matrons of experience recognise all this, and do their best to encourage their sisters and to cheer them on in what is really a very uphill job. No doubt none of us, much less a sister, ought to give way to the temptation to let things be. She is human, however, and there are sisters who do. Hence, the really good sister who perseveres to success deserves cordial recognition.''

Every matron of a hospital knows which of her sisters are careful in this matter of tirelessly battling against extravagance and waste. The chairman of every house committee should make it a practice to call upon the matron in his hospital each year to give him the names of those sisters whom she considers deserve encouragement. Then he will be in a position to secure that at the annual meeting, when the report is read in the presence of the governors, it will contain the names of those sisters who have been economical, accompanied by cheering words of acknowledgment and thanks for their successful endeavours. The chairman should also take care that a suitable bonus should be presented to each such sister as a token of appreciation for her services. If some such plan was universally followed, it would greatly cheer the recipients, and would act as an incentive to members of the nursing staff to persist in their endeavour to prevent waste. We are confident that such a course would result in the repayment of the bonuses many times over, by the increased economy which would soon be apparent throughout the administration of each hospital that adopts this system.

" How do you do? " The small pale face bore a welcoming smile, and the visitor to the ward smiled back at the child who thus greeted him. " I'm quite well," replied the visitor after a moment's pause, " but what about yourself? " " Oh, I'm getting on fine now, sir. I'm a bad run-over case. I was in the Sunday paper," she added with pride. " What made you come to the door to meet me? Did you expect to see a friend? " " Oh, no, sir; it's just sister's way; she always does it. When we're allowed to get up we copies sister. Our muvvers says it's learnt us good manners," and the visitor cordially agreed with the " muvvers."

⁂ It is our invariable practice to pay for all items of news which we may publish. The minimum payment is 5s. Paragraphs of about twenty lines in length—that is to say, of 150 words or less—are preferred. In this section during the war we propose to give 10s. to the correspondent who may send us in any week the best incident connected with hospital life and work. In this way we hope to afford to all practical aid and encouragement; while those who reciprocate will be able to render us invaluable service. Contributions should be addressed to The Editor, The Hospital, 28 and 29 Southampton Street, Strand, London, W.C., and, to be in time for the current week's issue, should reach him by the first post on Mondays.

### Mortuaries: Their Equipment and Management.

By A HOSPITAL MATRON.

